# Supplementary material for: Polar Solomon rings in ferroelectric nanocrystals
Source: Nat Commun. 2023 Jul 4;14:3941. doi: 10.1038/s41467-023-39668-y (PMC10319878; doi:10.1038/s41467-023-39668-y)
Supplement: Supplementary file 1 — Supplementary Information [file 41467_2023_39668_MOESM1_ESM.pdf]

Supplementary materials for

Polar Solomon Rings in Ferroelectric Nanocrystals

Jing Wang[1,2] †, Deshan Liang[1] †, Jing Ma[2] †, Yuanyuan Fan[1], Ji Ma[2,3], Hasnain Mehdi Jafri[1], Huayu Yang[1], Qinghua Zhang[4], Yue Wang[2], Changqing Guo[1], Shouzhe Dong[1], Di Liu[1], Xueyun Wang[5], Jiawang Hong[5], Nan Zhang[6], Lin Gu[2,4], Di Yi[2], Jinxing Zhang[7], Yuanhua Lin[2], Long-Qing Chen[8], Houbing Huang[1], Ce-Wen Nan[2]**

†*These authors contributed equally to this work.*

[*]E-mail: hbhuang@bit.edu.cn, cwnan@tsinghua.edu.cn.

This PDF file includes:

Supplementary Text

Supplementary Figures 1 to 22

Supplementary Table 1

Captions for Supplementary Movies 1 to 3

Other Supplementary Materials for this manuscript include the following:

Supplementary Movies 1 to 3

Supplementary text

The transformation between the 3D domain in a $BiFeO_3$ nanocrystal and the polar Solomon rings across a donut.

To have a deep understanding of how the domains in $BiFeO_3$ nanocrystals can be extracted to polar Solomon rings, we render $R_4^-[\bar{1}1\bar{1}]$, $R_3^+[\bar{1}\bar{1}1]$, $R_2^-[1\bar{1}\bar{1}]$, $R_1^+[111]$ polarization variants in red, and $R_4^+[1\bar{1}1]$, $R_3^-[11\bar{1}]$, $R_2^+[\bar{1}11]$, $R_1^-[\bar{1}\bar{1}\bar{1}]$ polarization variants in blue as shown in Supplementary Fig. 8a. When we gradually shrink the domain structure of the blue region and that of the red region into a tube shape, distorted Solomon rings are extracted, as shown in Supplementary Fig. 8b and 8c. Finally, standard Solomon rings are extracted in Supplementary Fig. 8d, where the two rings are interwoven across a donut. The reversible expanding and shrinking of the Solomon rings can be seen in Supplementary Movie 1.

The definition of linking number (LN) for polar Solomon rings.

Solomon rings are composed of two intertwined rings with four crossing points (Fig. 1a). Commonly, there are two overlapping possibilities for the intertwined rings at each crossing point, i.e., either the red ring is on top of the blue ring, or the blue ring is on top of the red ring. However, if the polar vector is further taken into consideration, there should be four possibilities at each crossing point (Supplementary Fig. 9a). The corresponding linking number for the crossing point is defined as n_1=+1, n_2=+1, n_3=-1, and n_4=-1, respectively. Thus, if the polar vector in both red ring and blue ring rotates counter-clockwise (Supplementary Fig. 9b and 9c) or clockwise (see Supplementary Fig. 9b and 9c from the backside of the paper), two kinds of polar Solomon rings with the respective linking number of +2 and -2 can be identified. Simultaneously, if the polar vector in the red ring rotates counter-clockwise whereas the polar vector in the blue ring rotates clockwise (Supplementary Fig. 9d and 9e) or vice versa (see Supplementary Fig. 9d and 9e from the backside of the paper), the corresponding linking number of the polar Solomon rings are -2 and +2, respectively. Thus, we conclude there are only two types of polar Solomon rings, e.g., with the linking number of +2 or -2.

Supplementary Figures

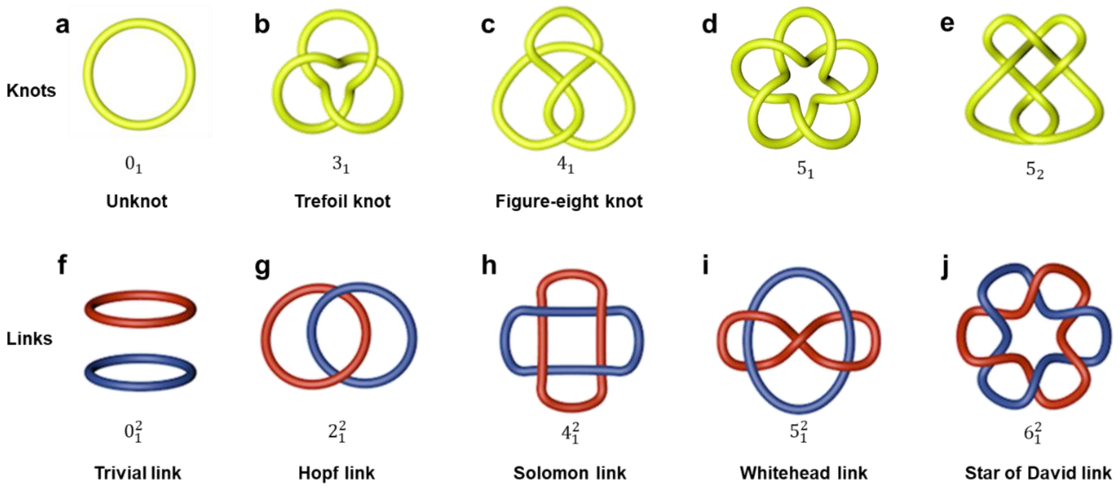

Fig. S1.

Stereoscopic representations of multiple knots and links. **a,** Unknot, 0_1. **b,** Trefoil knot, 3_1. **c,** Figure-eight knot, 4_1. **d,** 5_1 **e,** 5_2. **f,** Two-component trivial link, 0_1^2. **g,** Hopf link, 2_1^2. **h,** Solomon link, 4_1^2. **i,** Whitehead link, 5_1^2 **j,** Star of David link, 6_1^2.

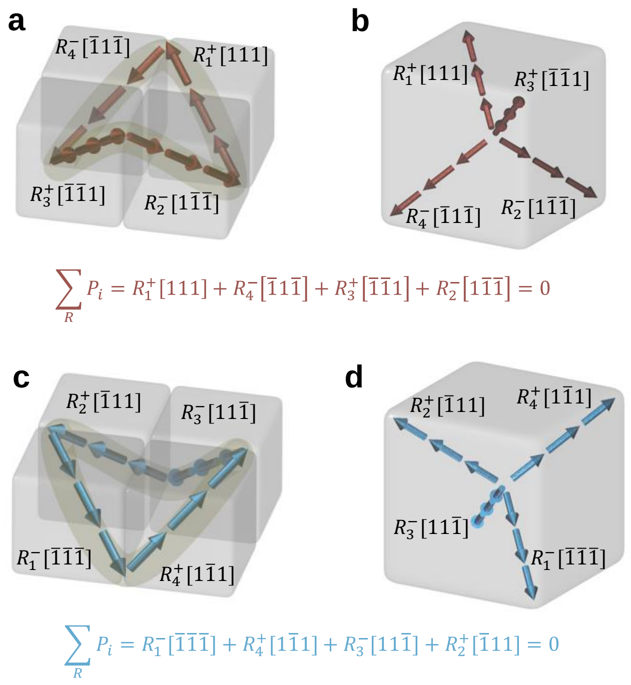

Fig. S2.

Decoupled Solomon rings composed of 3D vortex with rhombohedral ferroelectric polarization variants. **a,** A 3D polar vortex composed of $R_4^-[\bar{1}1\bar{1}]$, $R_3^+[\bar{1}\bar{1}1]$, $R_2^-[1\bar{1}\bar{1}]$, $R_1^+[111]$ polarization variants in rhombohedral phase $BiFeO_3$. **b,** Rearrangement of the corresponding polarization variants in A from one original point. **c,** A 3D polar vortex composed of $R_4^+[1\bar{1}1]$, $R_3^-[11\bar{1}]$, $R_2^+[\bar{1}11]$, $R_1^-[\bar{1}\bar{1}\bar{1}]$ polarization variants in rhombohedral phase $BiFeO_3$. **d,** Rearrangement of the corresponding polarization variants in C from one original point. The selected polarization variants for one 3D vortex follow the polarization conservation law.

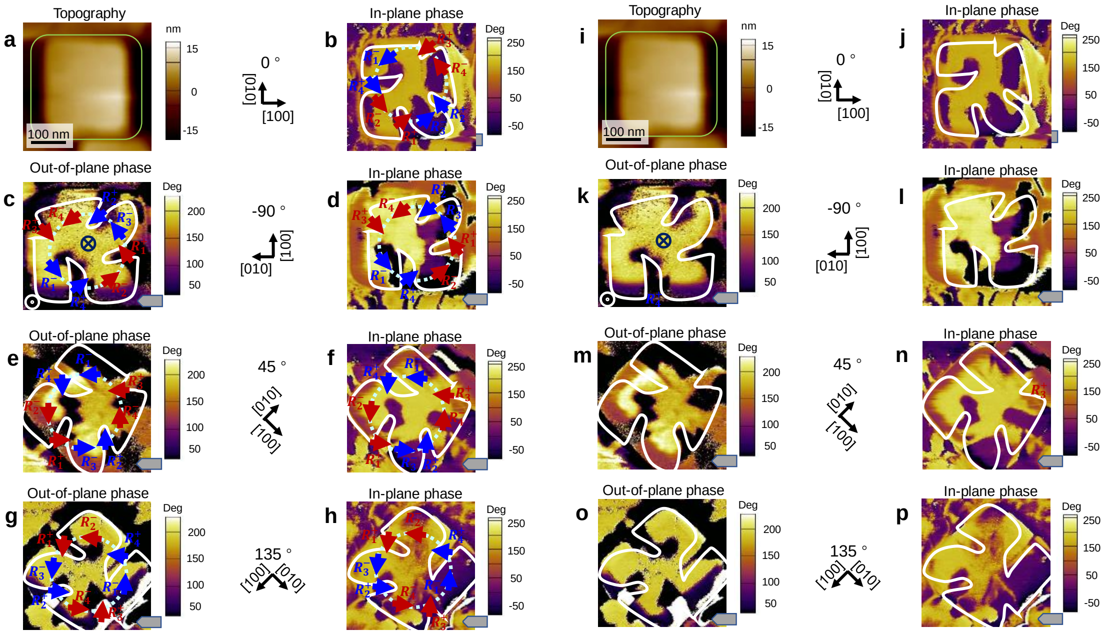

Fig. S3.

PFM characterization of the OOP and IP polarization projection for polar Solomon rings with LK of +2. **a, b,** Morphology and IP PFM phase image for the $BiFeO_3$ nanocrystal at the initial state. **c-h,** OOP (**c**, **e**, **g**) and IP (**d**, **f**, **h**) PFM phase images for the $BiFeO_3$ nanocrystal when the specimen is rotated by -90° (**c**, **d**), 45° (**e**, **f**), and 135° (**g**, **h**) with respect to the cantilever. **i-p,** The corresponding raw data. This $BiFeO_3$ nanocrystal is the same as presented one in Fig. 1D in the main text.

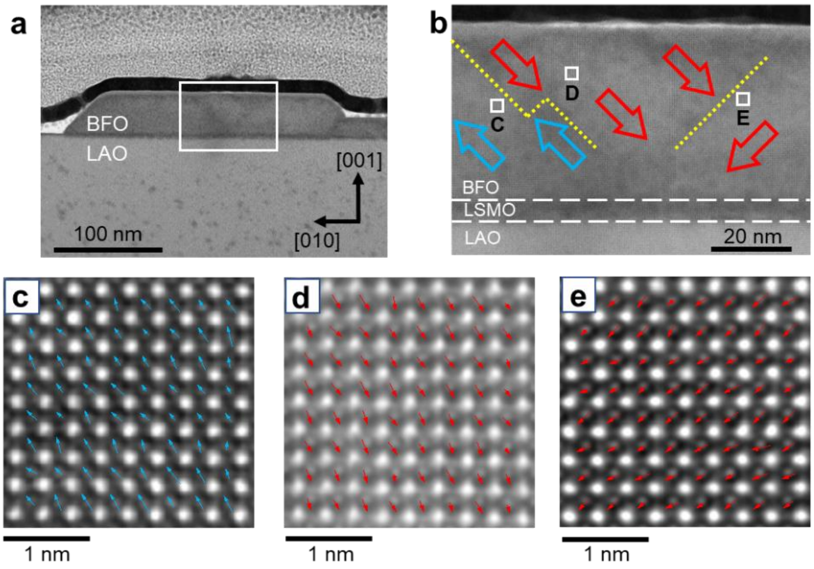

Fig. S4.

Cross-sectional polarization distribution of $BiFeO_3$ nanocrystal. **a,** Cross-sectional topography for an entire nanocrystal. **b,** Polarization projections in (100)-plane for the magnified region enlaced by white box in (**a**). **c-e,** Atomic polar mapping for three different domain regions highlighted by white boxes in (**b**), respectively.

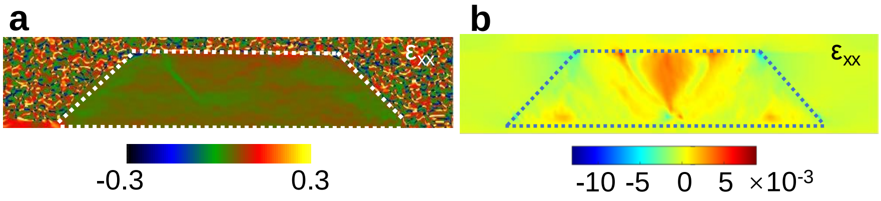

Fig. S5.

Cross-sectional strain distribution of a BFO nanocrystal. **a,** GPA image for the cross-sectional BFO nanocrystal. **b,** Phase-field simulations for strain distribution in a BFO nanocrystal.

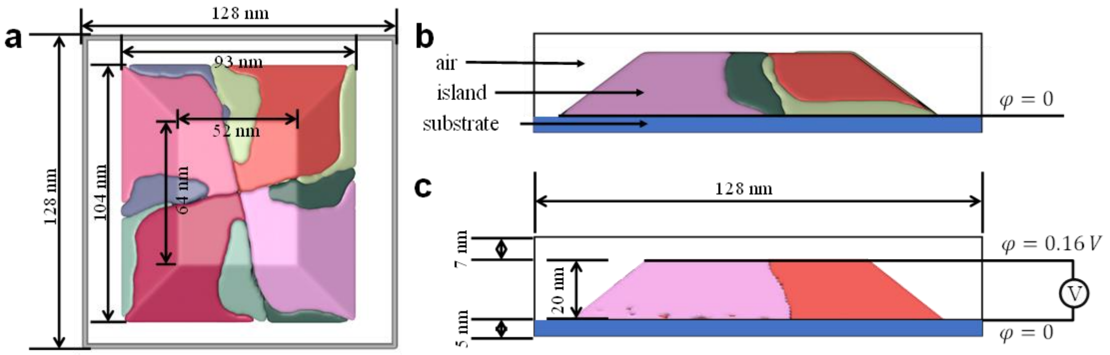

Fig. S6.

Island boundary conditions used in phase-field simulations. **a,** The dimensions of the island. The island boundary conditions for **b,** Solomon rings and **c,** quad-domains.

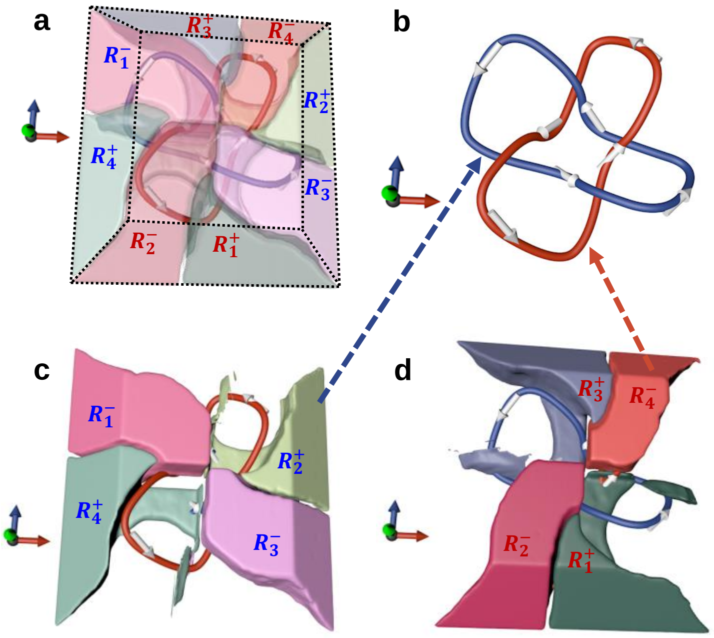

Fig. S7.

Illustration of the interwoven way for polar Solomon rings with LK of +2 by phase-field simulations. **a,** The 3D domain structure with nested polar Solomon rings. **b,** The interwoven red ring and blue ring with counterclockwise polarization rotation extracted from the 3D domain pattern in (a). The white arrows indicate the four polarization variants for each ring. **c,** Polar Solomon rings with the red ring nested in polarization variants of $R_4^+[1\bar{1}1]$, $R_3^-[11\bar{1}]$, $R_2^+[\bar{1}11]$ and $R_1^-[\bar{1}\bar{1}\bar{1}]$, e.g., the blue ring. **d,** Polar Solomon rings with the blue ring nested in polarization variants of $R_4^-[\bar{1}1\bar{1}]$, $R_3^+[\bar{1}\bar{1}1]$, $R_2^-[1\bar{1}\bar{1}]$ and $R_1^+[111]$, e.g., the red ring.

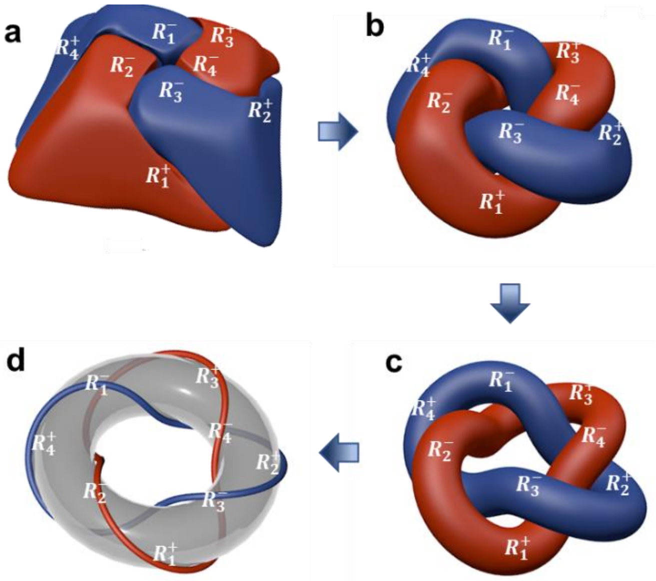

Fig. S8.

The transformation from a 3D domain to polar Solomon rings. **a-c,** Evolution of the 3D domain pattern in $BiFeO_3$ nanocrystals by gradually shrinking each domain. **d,** Extracted polar Solomon rings across a donut.

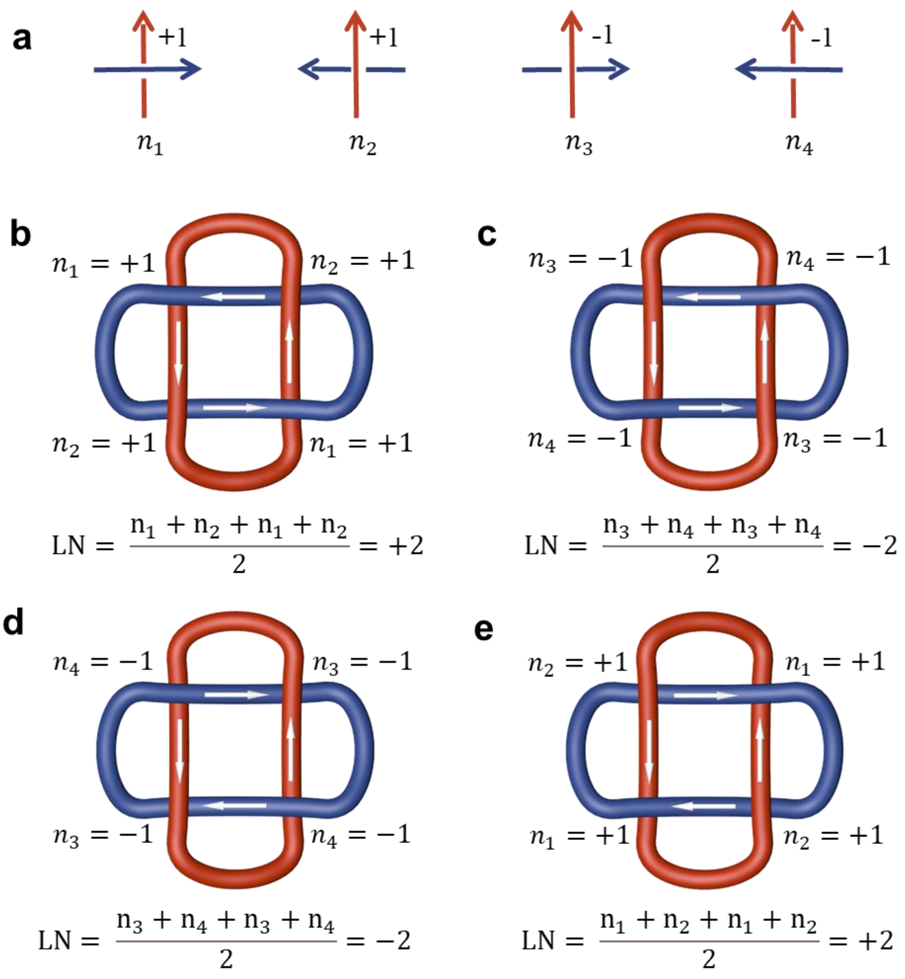

Fig. S9.

Mathematical definition of polar Solomon rings. **a,** The illustration of each intersecting knot for n_1, n_2, n_3, and n_4. **b, c,** Polar Solomon rings with calculated LN of +2 and -2, where the polarization in both red ring and blue ring rotates counterclockwise. **d, e,** Polar Solomon rings with calculated LN of -2 and +2, where the polarization in the red ring rotates counterclockwise and the polarization in the blue ring rotates clockwise.

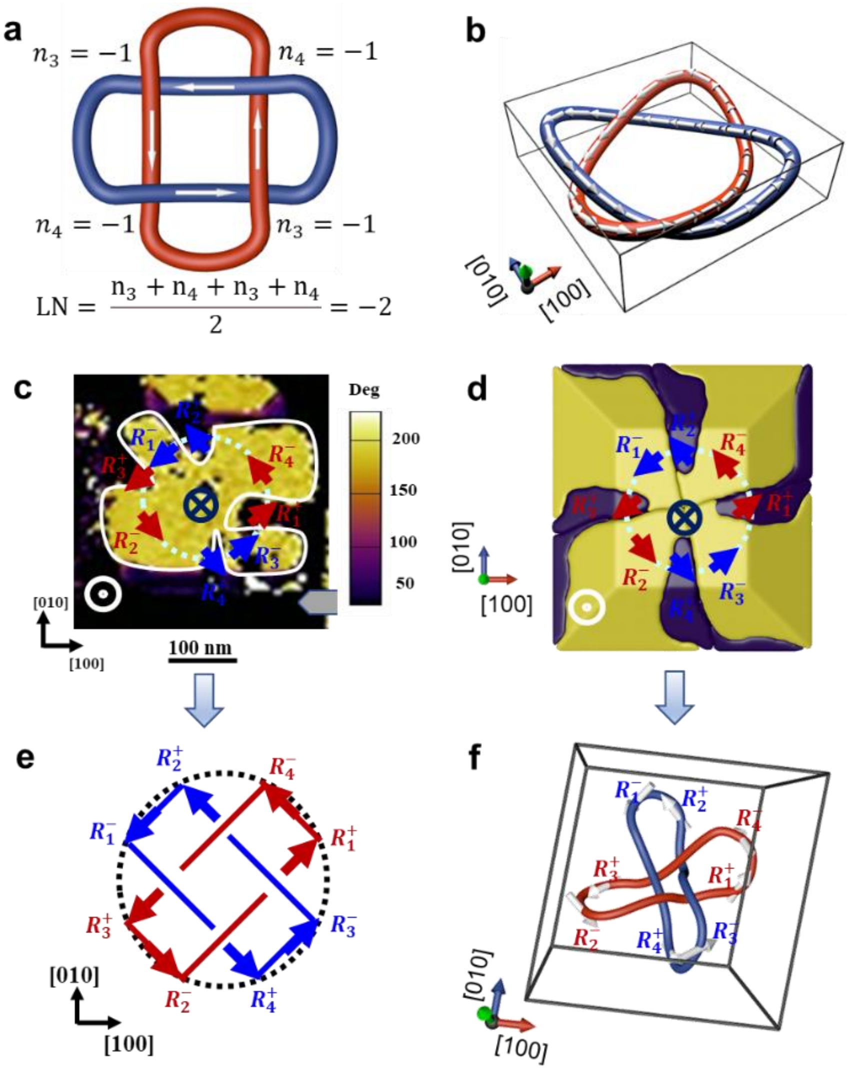

Fig. S10.

Observation of polar Solomon rings with LK of -2 in $BiFeO_3$ nanocrystal. **a,** The definition of polar Solomon rings with LK of -2. **b,** The distorted 3D polar Solomon rings with LK of -2. **c,** The construction of OOP and IP polarization projection for a $BiFeO_3$ nanocrystal by PFM measurement. **d,** Phase-field simulations of the 3D domain pattern for $BiFeO_3$ nanocrystal. **e,** The extracted two intersecting polar vortices projected in (001)-plane. **f,** The extracted polar Solomon rings from (**d**).

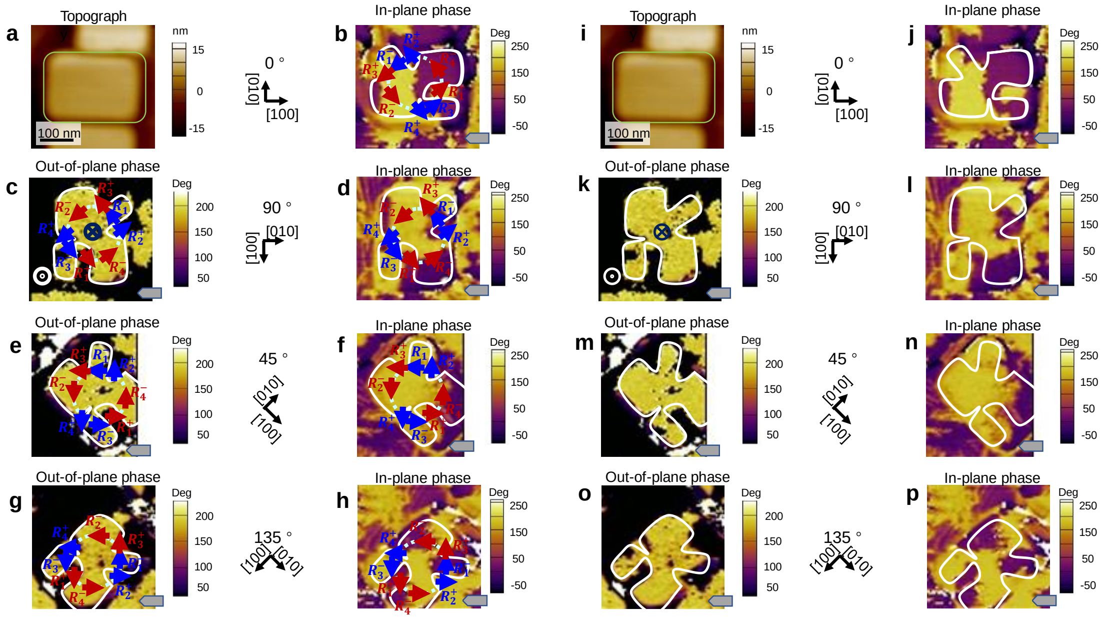

Fig. S11.

PFM characterization of the OOP and IP polarization projection for polar Solomon rings with LK of -2. This $BiFeO_3$ nanocrystal is the same as the presented one in Fig. S8. **a, b,** Morphology and IP PFM phase image for the $BiFeO_3$ nanocrystal at the initial state. **c-f**, OOP (**c**, **e**, **g**) and IP (**d**, **f**, **h**) PFM phase images for the $BiFeO_3$ nanocrystal when the specimen is rotated by 90° (**c**, **d**), 45° (**e**, **f**), and 135° (**g**, **h**) with respect to the cantilever. **i-p,** The corresponding raw data.

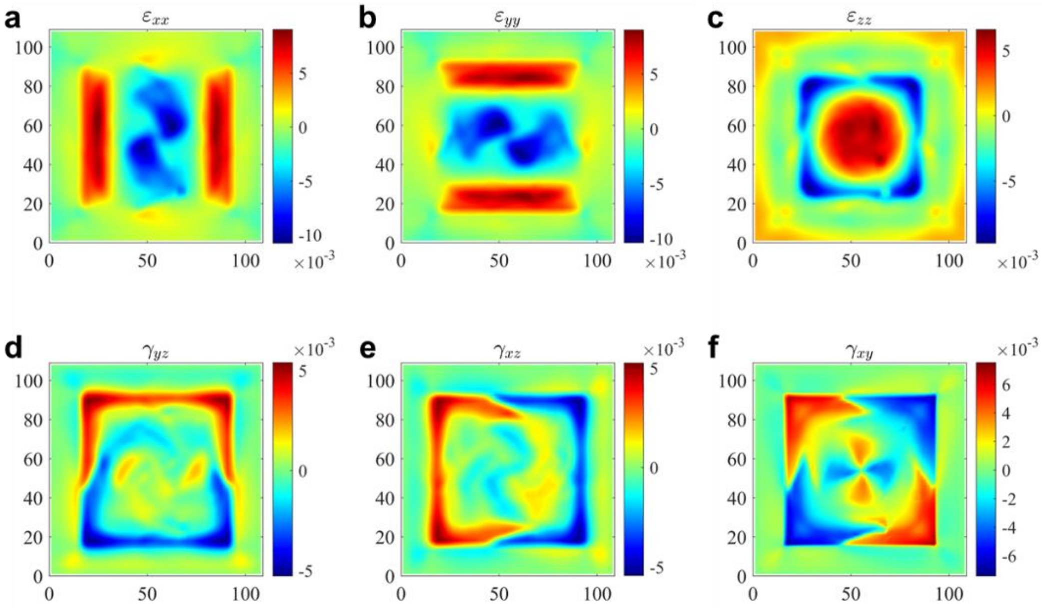

Fig. S12.

Strain distribution in $BiFeO_3$ nanocrystal with polar Solomon rings. **a,** ε_{xx}. **b,** ε_{yy}. **c,** ε_{zz}. **d,** γ_{yz}. **e,** γ_{xz}. **f,** γ_{xy}.

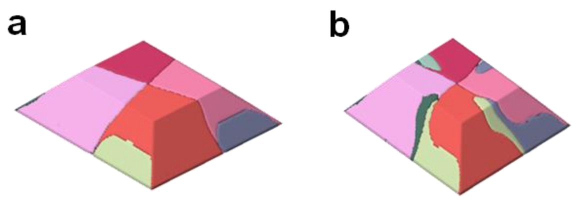

Fig. S13.

Phase-field simulations **a,** without and **b,** with consideration of depolarization field.

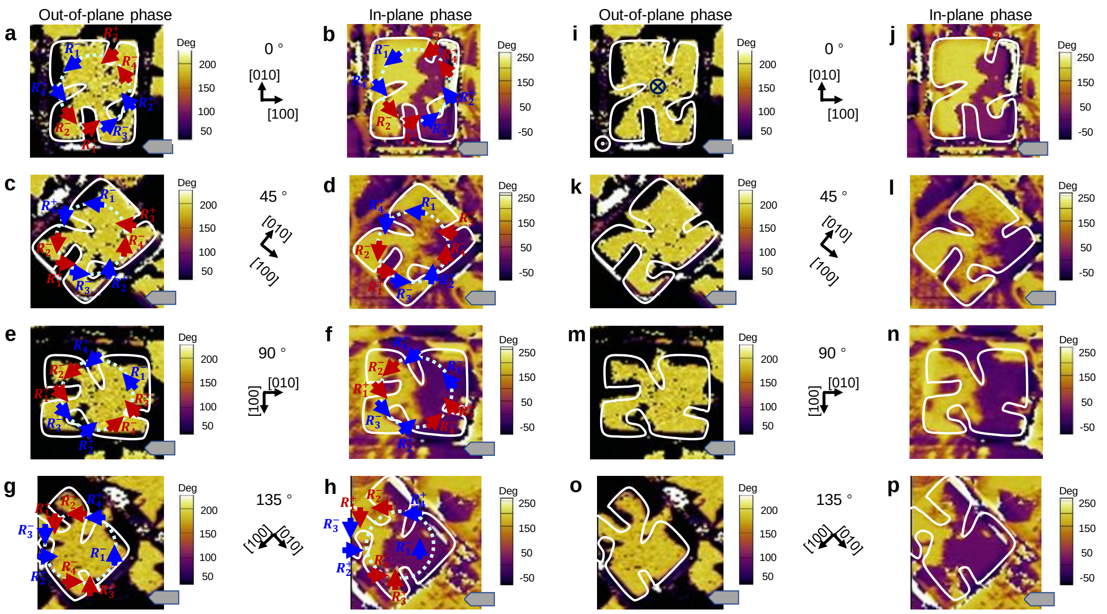

Fig. S14.

PFM characterization of the OOP and IP polarization projection for polar Solomon rings with LK of +2. **a, b,** OOP (**a**) and IP (**b**) PFM phase images for the $BiFeO_3$ nanocrystal in the initial state. **c, d,** OOP (**c**) and IP (**d**) PFM phase images for the $BiFeO_3$ nanocrystal when the specimen is rotated by 45° with respect to the cantilever. **e, f,** OOP (**e**) and IP (**f**) PFM phase images for the $BiFeO_3$ nanocrystal when the specimen is rotated by 90° with respect to the cantilever. **g, h,** OOP (**g**) and IP (**h**) PFM phase images for the $BiFeO_3$ nanocrystal when the specimen is rotated by 135° with respect to the cantilever. **i-p,** The corresponding raw data.

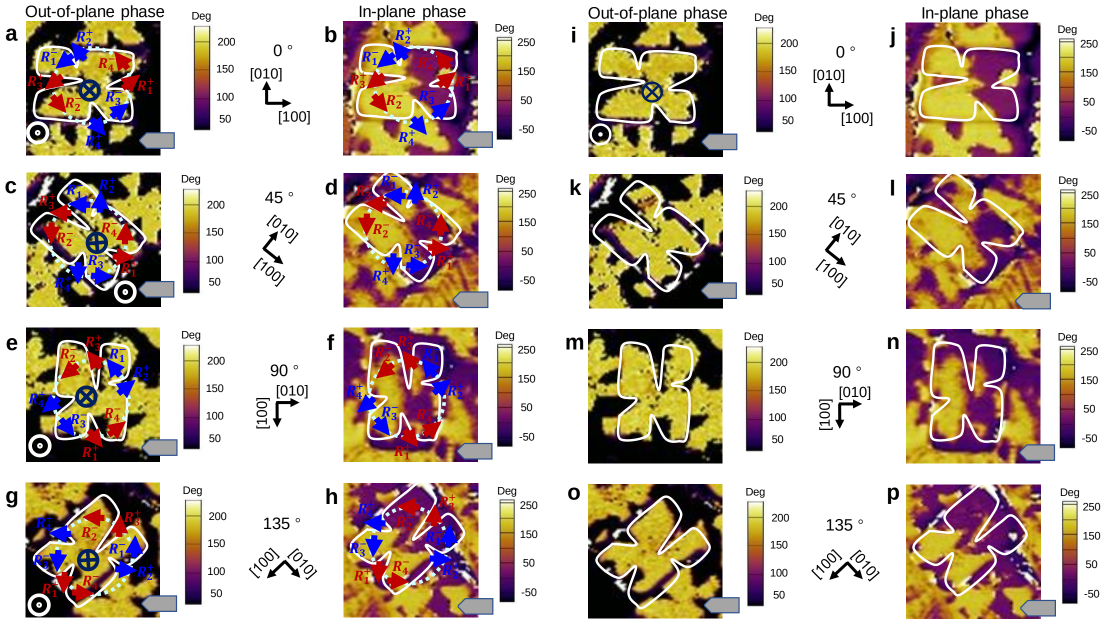

Fig. S15.

PFM characterization of the OOP and IP polarization projection for Solomon rings with LK of -2. **a, b,** OOP (**a**) and IP (**b**) PFM phase images for the $BiFeO_3$ nanocrystal in the initial state. **c, d,** OOP (**c**) and IP (**d**) PFM phase images for the $BiFeO_3$ nanocrystal when the specimen is rotated by 45° with respect to the cantilever. **e, f,** OOP (**e**) and IP (**f**) PFM phase images for the $BiFeO_3$ nanocrystal when the specimen is rotated by 90° with respect to the cantilever. **g, h,** OOP (**g**) and IP (**h**) PFM phase images for the $BiFeO_3$ nanocrystal when the specimen is rotated by 135° with respect to the cantilever. **i-p,** The corresponding raw data.

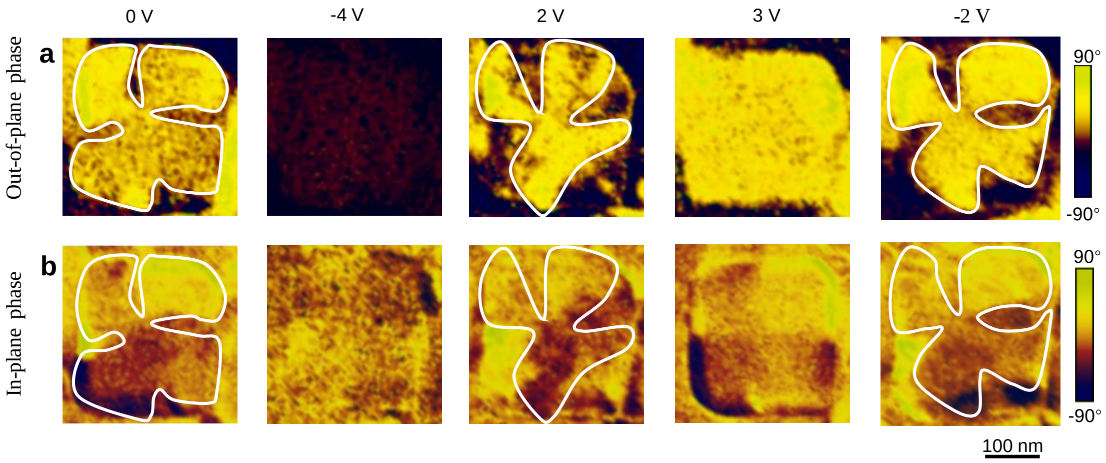

Fig. S16.

Electric-field driven topological phase transition between polar Solomon rings and vertex structures in a $BiFeO_3$ nanocrystal. **a, b,** OOP (**a**) and IP (**b**) PFM phase images for the domain pattern in a $BiFeO_3$ nanocrystal when the electric fields of 0 V, -4 V, 2 V, 3 V, and -2 V are applied to the scanning probe, respectively. This is the corresponding raw data related to Fig. 3a, b in the main text.

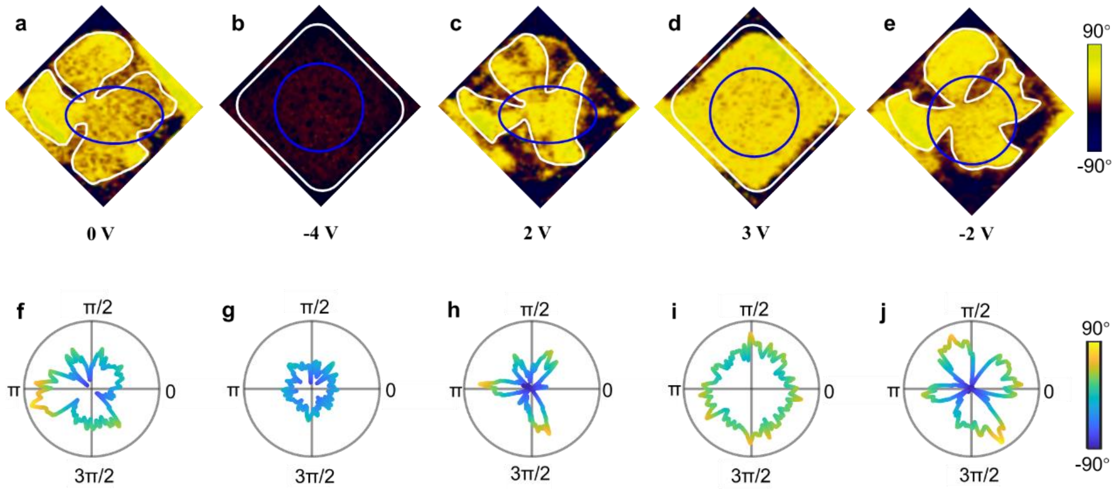

Fig. S17.

Electric-field control of the polar Solomon rings in $BiFeO_3$ nanocrystal. **a-e,** OOP PFM phase image when the nanocrystal is composed to 0V, -2V, -4V, 2V and 3V probe voltage, respectively. **f-j** The corresponding line profile of the OOP phase along the blue circle in (**a-e**), respectively.

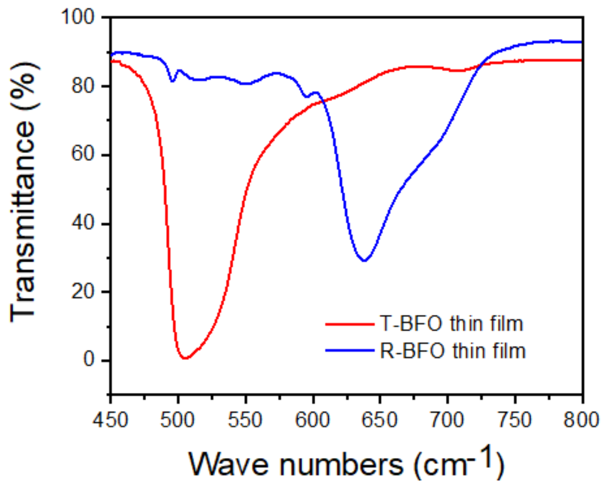

Fig. S18.

FT-IR spectrum for tetragonal $BiFeO_3$ (T-BFO) and rhombohedral $BiFeO_3$ (R-BFO) thin film.

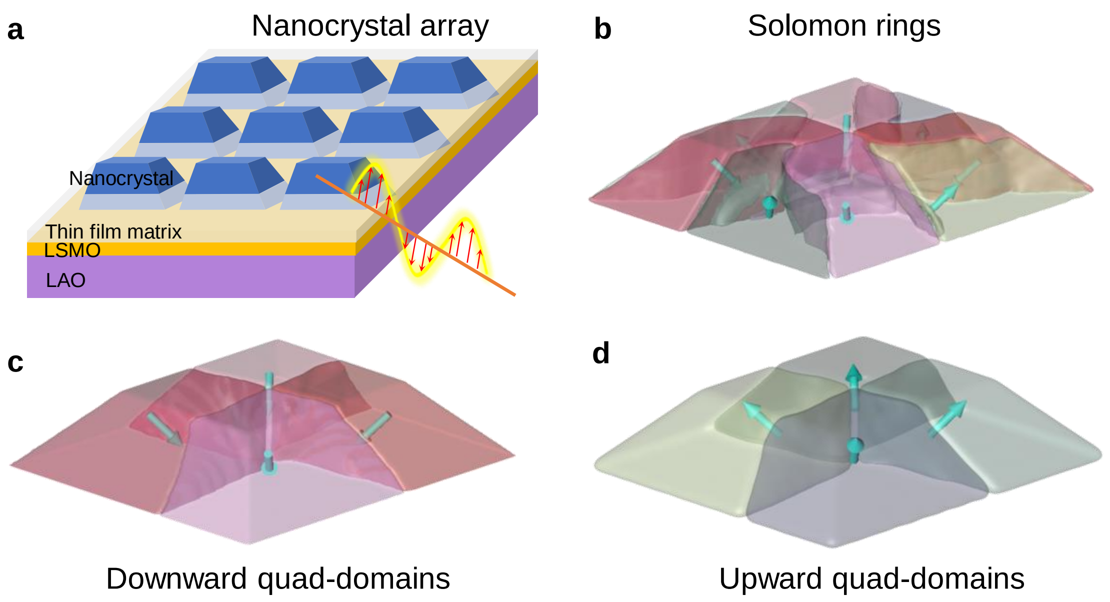

Fig. S19.

Illustration of the relative direction between polarized light and the topological domain structures. **a,** Schematic diagram of $BiFeO_3$ nanocrystal array and the polarized light. **b-d,** Illustration of the polarization direction for Solomon rings (**b**), downward quad-domains (**c**) and upward quad-domains (**d**).

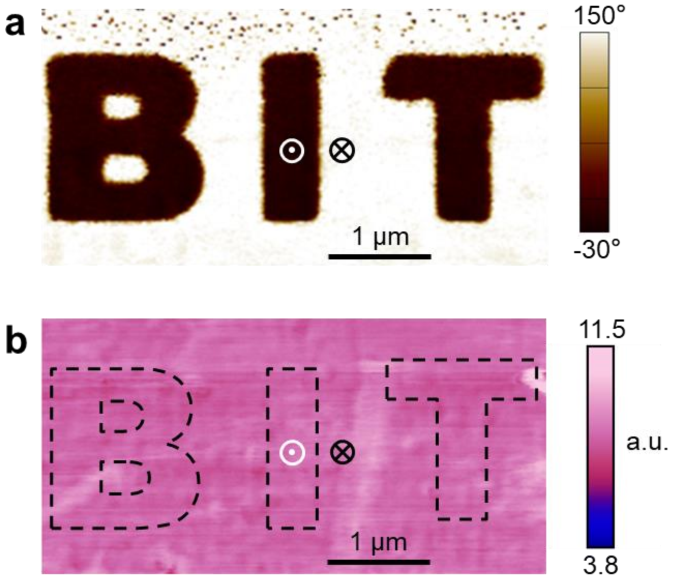

Fig. S20.

Similar IR absorption for upward and downward domains in $BiFeO_3$ continuous thin film. **a,** OOP phase image for the written 'BIT' characteristics. **b,** The corresponding IR absorption for the same location. The result indicates the similar IR absorption intensity for the upward and downward domain in the continuous $BiFeO_3$ thin film.

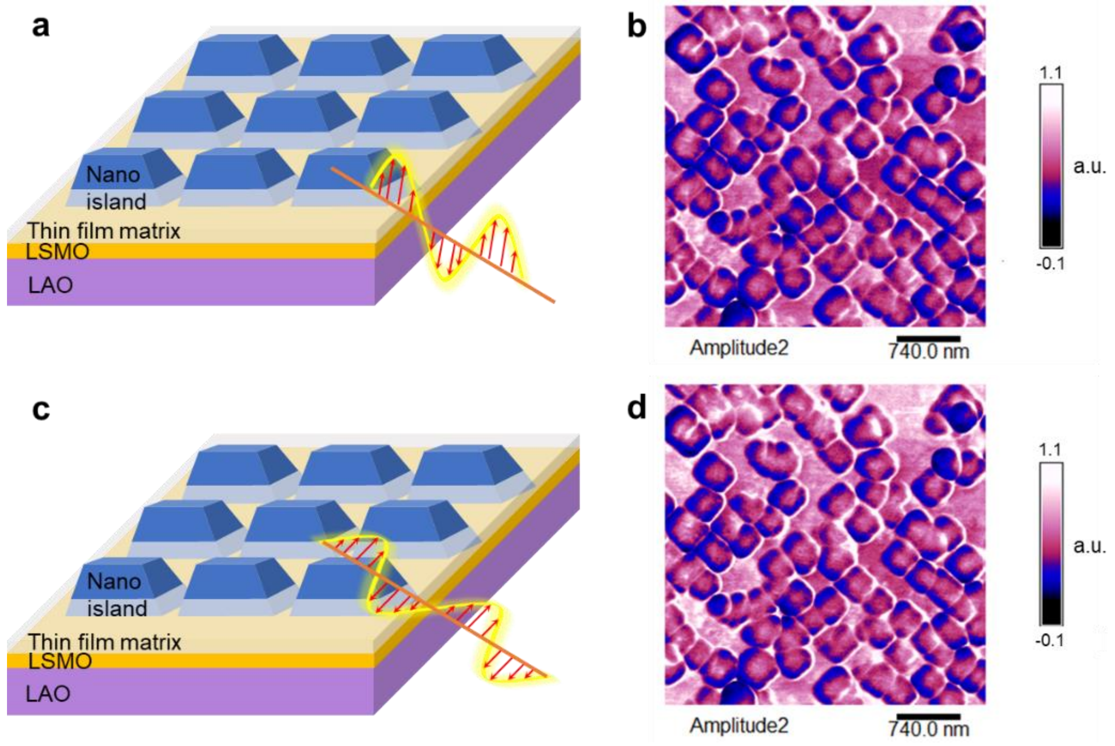

Fig. S21.

AFM IR absorption for Solomon rings with OOP and IP electric field vector. **a,c,** Schematic for the experimental setup with OOP (**a**) and IP (**c**) electric field vector. **b, d,** The corresponding IR absorption image when the electric field is OOP (**b**) and IP (**d**).

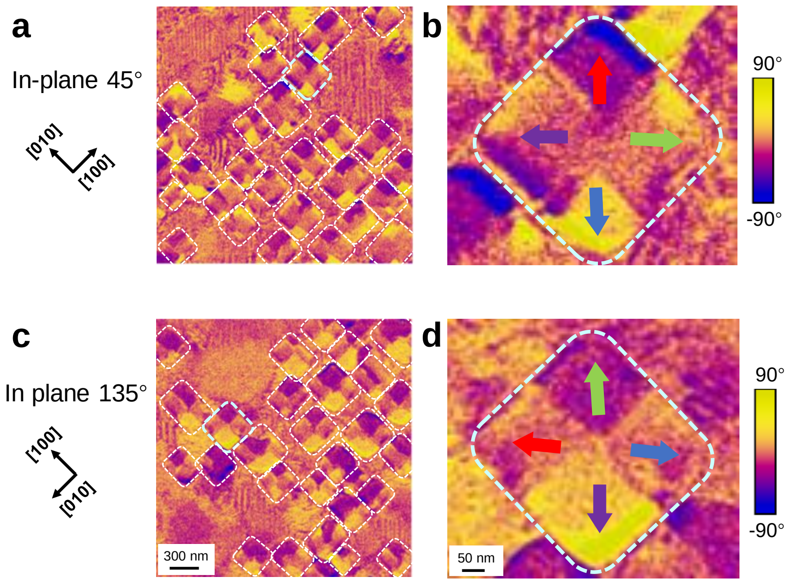

Fig. S22.

In-plane PFM phase image for $BiFeO_3$ nanocrystals after poling. **a, b,** A large scale (**a**) and magnified (**b**) IP PFM phase image for the upward quad-domains when the sample is positioned at 45°with respect to the cantilever. **c, d,** A large scale (**c**) and magnified (**d**) IP PFM phase image for the upward quad-domains when the sample is positioned at 135°with respect to the cantilever.

Supplementary Table 1.

List of coefficients used in the present work.

Coefficients	Values
α_1	4.64385×(T-1103)×10^5 $C^{-2}m^2N$
α_{11}	2.29047×10^8 $C^{-4}m^6N$
α_{12}	3.06361×10^8 $C^{-4}m^6N$
α_{111}	5.99186×10^9 $C^{-6}m^{10}N$
α_{112}	-3.33980×10^8 $C^{-6}m^{10}N$
α_{123}	-1.77754×10^9 $C^{-6}m^{10}N$
Q_{11}	3.2×10^{-2} $C^{-2}m^4$
Q_{12}	-1.6×$10^{-2}$$C^{-2}m^4$
Q_{44}	2.0×10^{-2} $C^{-2}m^4$
C_{11}	302 GPa
C_{12}	162 GPa
C_{44}	68 GPa
