# Supplementary material for: Polar Solomon rings in ferroelectric nanocrystals
Source: Nat Commun. 2023 Jul 4;14:3941. doi: 10.1038/s41467-023-39668-y (PMC10319878; doi:10.1038/s41467-023-39668-y)
Supplement: Supplementary file 3 — Description of Additional Supplementary Files [file 41467_2023_39668_MOESM3_ESM.pdf]

Description of Additional Supplementary Files

File Name: Supplementary Movie 1
Description: The transformation process between the 3D domain and the polar Solomon rings.

File Name: Supplementary Movie 2
Description: The dynamic domain evolution from polar Solomon rings to upward quad-domains.

File Name: Supplementary Movie 3
Description: The dynamic domain evolution from polar Solomon rings to downward quad-domains.
